# Ultrasound-Assisted Extraction of Bioactive Compounds from Broccoli By-Products

**DOI:** 10.3390/foods13101441

**Published:** 2024-05-07

**Authors:** Lorena Martínez-Zamora, Seyedehzeinab Hashemi, Marina Cano-Lamadrid, María Carmen Bueso, Encarna Aguayo, Mathieu Kessler, Francisco Artés-Hernández

**Affiliations:** 1 Postharvest and Refrigeration Group, Department of Agricultural Engineering & Institute of Plant Biotechnology, Universidad Politécnica de Cartagena, 30203 Cartagena, Murcia, Spain; lorena.martinez23@um.es (L.M.-Z.); seyedehzeinab.hashemi@edu.upct.es (S.H.); marina.cano@upct.es (M.C.-L.); encarna.aguayo@upct.es (E.A.); 2 Department of Food Technology, Nutrition, and Food Science, Faculty of Veterinary Sciences, University of Murcia, 30071 Espinardo, Murcia, Spain; 3 Department of Applied Mathematics and Statistics, Universidad Politécnica de Cartagena, 30202 Cartagena, Murcia, Spain; mcarmen.bueso@upct.es (M.C.B.); mathieu.kessler@upct.es (M.K.)

**Keywords:** *Brassica oleracea*, circular economy, food loss, green technologies isothiocyanates, kinetic modeling, phytochemicals, revalorization

## Abstract

The objective of this work was to gain insight into the operating conditions that affect the efficiency of ultrasound-assisted extraction (UAE) parameters to achieve the best recovery of bioactive compounds from broccoli leaf and floret byproducts. Therefore, total phenolic content (TPC) and the main sulfur bioactive compounds (sulforaphane (SFN) and glucosinolates (GLSs)) were assayed. Distilled water was used as solvent. For each byproduct type, solid/liquid ratio (1:25 and 2:25 g/mL), temperature (25, 40, and 55 °C), and extraction time (2.5, 5, 7.5, 10, 15, and 20 min) were the studied variables to optimize the UAE process by using a kinetic and a cubic regression model. TPC was 12.5-fold higher in broccoli leaves than in florets, while SFN was from 2.5- to 4.5-fold higher in florets regarding the leaf’s extracts obtained from the same plants, their precursors (GLS) being in similar amounts for both plant tissues. The most efficient extraction conditions were at 25 °C, ratio 2:25, and during 15 or 20 min according to the target phytochemical to extract. In conclusion, the type of plant tissue and used ratio significantly influenced the extraction of bioactive compounds, the most efficient UAE parameters being those with lower energy consumption.

## 1. Introduction

Broccoli is the most widely consumed vegetable from the *Brassicaceae* family, reaching a worldwide production of 25.5 M t per year [1]. However, just 9–10% of the total biomass of the plant is consumed, mainly comprising their florets [2]. Hence, abundant byproducts are generated after harvest, particularly leaves and stalks, but also the non-edible florets. Broccoli leaf byproducts contain higher levels of total phenolic content (TPC), total antioxidant capacity (TAC), chlorophylls, and vitamins (E and K) than florets or stalks [3]. A great interest in broccoli byproducts is focused on their primary bioactive compounds, the glucosinolates (GLSs), which serve as precursors for potent anticarcinogens and antimutagens such as sulforaphane (SFN) [4,5]. It is worth noting that brassica vegetables contain a predominant amount of GLS, and their accumulation is influenced by several factors such as genotype, variety, cultivation conditions, developmental stage, type of plant tissue, and postharvest handling conditions [6].

Recent studies have focused on valorizing waste from different brassica vegetables, including broccoli leaves and stalks, cabbage, and cauliflower leaves and florets [7,8,9,10]. Several researchers highlighted the potential of extracting bioactive compounds and nutrients using both conventional and environmentally friendly technologies [11,12,13,14].

Ultrasound-assisted extraction (UAE) offers the advantage of reducing solvent usage, energy consumption, and extraction time, making it an eco-friendly and cost-effective method [15]. The frequency range of UAE equipment used varies from 20 to 50 kHz, while the power units depend on specific equipment and report values between 100 and 500 W, 50 W L^−1^, or 0.228 W cm^−2^. The most favorable results are achieved using an aqueous solvent, as was selected for the present study. Water serves as the extractant, single or combined with other organic solvents [3], the most used being ethanol, methanol, or acetonitrile [16,17,18,19].

Liu et al. [7] recently reported a more efficient SFN extraction with a water-to-material ratio of 1:10 compared to 1:50 for ethyl-acetate. The solid-to-liquid ratio (g/mL) in most studies ranged between 1:2 and 1:50, with just one study using a more diluted extract (0.06:30) [20]. The extraction temperature depends on the specific target compound or desired function to be achieved through the extraction. Temperatures above 45 °C were mainly used for the extraction of phenolic compounds [15,19], while temperatures below 30 °C were found to be most effective for GLS and SFN extraction [18,19,21]. In this sense, broccoli leaves have also been shown to be source of GLS, as identified by Ares et al. [22], although at a lower concentration than other broccoli tissues [23]. Also, other technologies such as pulsed electric fields have been useful in optimizing the extraction of bioactive compounds [24]. Furthermore, a short microwave-assisted extraction pretreatment, which increased the temperature, favored SFN extraction due to the inactivation of the myrosinase enzymes and GLS-SFN conversion.

However, there is a lack of important information concerning the optimal UAE conditions to achieve the highest bioactive compound extraction [2]. Therefore, the novelty and aim of this study was to study the effect of essential operating conditions for the optimal aqueous UAE of the main phytochemicals in broccoli floret and leaf byproducts: (i) byproduct/solvent ratio; (ii) temperature; and (iii) time. As key biocompounds, glucosinolates, isothiocyanates, and total phenolic content were assayed. Regarding previous studies, two models were considered to elucidate the results obtained in the present work. The Peleg model, which is a kinetic model, and an empirical cubic regression model were fitted to experimental data over time, under fixed conditions of temperature and ratio. 

## 2. Materials and Methods

### 2.1. Vegetal Material and Experimental Design

Broccoli leaves and discarded florets (Naxos F1 Hybrid Broccoli, SAKATA SEED IBÉRICA SLU, Alboraya, Spain) were supplied by Grupo Lucas^®^ (Murcia, Spain) in November 2022. Drying and grinding (<56 µm) pretreatments were carried out to obtain a stable and homogeneous raw material for extraction. Samples were freeze-dried using a Telstar^®^ LyoBeta (Terrassa, Spain) freeze-dryer. Once both broccoli byproducts were obtained, they presented a moisture content (measured following a standardized method [25]) of 8.80 ± 0.40% in the case of broccoli leaves and 12.36 ± 0.36% for broccoli florets (Table 1). Broccoli stem/stalk byproducts were firstly used as another potential source of phytochemicals. Nevertheless, this part was not included in this experiment due to its high fiber content, which did not allow for milling the samples to the target size, and the high enzymatic activity inducing relevant browning after freeze-drying. Broccoli tissues used in the present experiment were characterized according to the methodology described in Section 2.3.

### 2.2. Ultrasound Extraction of Bioactive Compounds

**Figure 1:**
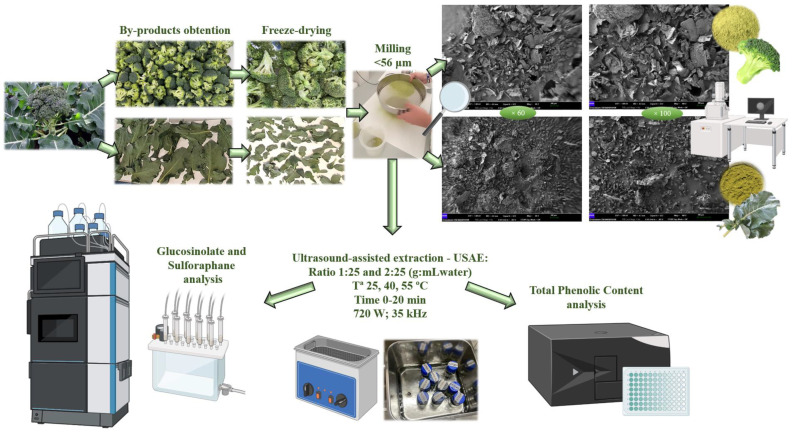
Experimental design. Original SEM pictures are shown in Figure S1.

The extraction of bioactive compounds was carried out using a Sonorex^®^ Digiplus DL 514 BH ultrasound bath (Berlin, Germany) with a capacity of 18.7 L, using a power of 720 W, at a frequency of 35 kHz at set temperature. The particle size was <56 µm and distilled water was used as solvent. The process was continuously conducted without pulses. The studied variables were (i) part of the broccoli (florets and leaves), (ii) solid/liquid ratio (1:25 g/mL (R1) and 2:25 g/mL (R2), (iii) temperature (25, 40, and 55 °C), and (iv) time (0, 2.5, 5, 7.5, 10, 15, and 20 min). Once the extraction was completed, samples were filtered to separate the solid from the extract. Part of the samples were filtered with a 0.45 µm filter for subsequent analysis by liquid chromatography. The extracts were stored at −80 °C until analysis. The experimental design is shown in Figure 1.

### 2.3. Bioactive Compounds Analysis

The in vitro total antioxidant capacity (TAC) of the broccoli tissues studied was assessed for its characterization by three methods: the ferric reducing antioxidant potential (FRAP) [26], the 2,2-diphenyl-1-picrylhydrazyl (DPPH) radical scavenging assay [27], and the 2,2′-azino-bis(3-ethylbenzothiazoline-6-sulfonate) radical cation (ABTS^•+^) [28]. The absorbance was measured at 495, 515, and 414 nm, respectively, using a microplate reader (Tecan Infinite M200, Männedorf, Switzerland). Results were expressed in g Trolox Equivalent (TE) kg^−1^ dw. We used the following standard curves: y = 7.8538x + 0.0453; R^2^ = 0.9972 for FRAP; y = 4.2561x + 0.1003; R^2^ = 0.9859 for DPPH; and y = 12.188x + 0.0156; R^2^ = 0.9941 for ABTS.

Total phenolic content (TPC) of broccoli tissues and broccoli extracts studied was determined as previously described [29]. Briefly, a 19 μL sample previously extracted was placed on a 96-well plate (Greiner Bio-One; Frickenhausen, Germany) and mixed with 29 μL of 1 mol L^−1^ Folin–Ciocalteu reagent. After 3 min incubation in darkness, 192 μL of 0.4% Na_2_CO_3_ + 2% NaOH solution was added. The absorbance was measured 1 h later (incubated in darkness) at 750 nm using a microplate reader (Tecan Infinite M200, Männedorf, Switzerland). TPC was expressed as g gallic acid equivalents (GAE) kg^−1^ (dw) using a standard curve (y = 0.2457x − 0.0294; R^2^ = 0.9906). 

The extraction of desulfoglucosinolates and identification of individual GLS content was carried out following the method described by Castillejo et al. [30] to characterize the studied broccoli tissues and after its US extraction. Immediately after the extraction, GLSs were de-sulfated and purified using disposable polypropylene columns (Thermo Fisher Scientific, Waltham, MA, USA). Three mL of clarified ethanolic extract was added into a prepared column and allowed to drip through slowly. Columns were washed with water followed by 0.02 M sodium acetate. Purified sulfatase (75 μL) was added to each sample and left at room temperature overnight during 16 h. Desulfoglucosinolates were eluted with 1.25 mL of water and kept at −80 °C after 0.2 µm filtration until U-HPLC analysis. An ultrahigh-performance liquid chromatography (UHPLC) device (Shimadzu, Kyoto, Japan) equipped as described by Castillejo et al. [30] was used. Separation of desulfoglucosinolates in the UHPLC system was achieved using water (A) and acetonitrile (B) as mobile phases with a flow rate of 1.5 mL min^−1^ and a gradient of 0/100, 28/80, 30/100 (min/%; A) with an injection volume of 20 μL sample. Desulfoglucosinolates were detected at 227 nm. The main individual GLS identified and quantified were (i) GLF: dsf-glucoraphanin (accounted the ~35% of the total glucosinolate content); and (ii) GLB: dsf-glucobrassicin (accounted the ~50% of the total glucosinolate content). Total glucosinolate content (ΣGL) was calculated as the sum of both compounds and the results were expressed as g kg^−1^ dw. Glucoraphanin (PhytoLab GmbH & Co, Germany) was used as a standard to quantify (y = 4 × 10^7^ − 5377.3; R^2^ = 0.9952; b = 35,814,500; LOD: 0.0036 mg mL^−1^; LOQ: 0.0119 mg mL^−1^).

Sulforaphane (SFN) was extracted and analyzed to characterize broccoli tissues studied and after its US extraction following the method previously described by Castillejo et al. [30]. For that, 5 μL sulforaphane extracts were injected in a U-HPLC (Shimadzu, Kyoto, Japan) equipped as described in the method with a Gemini C18 column (250 mm × 4.6 mm, 5 µm particle size; Phenomenex, Torrance CA, USA). The mobile phases used were 0.02 mol L^−1^ ammonium formate (A) and acetonitrile (B) with a 0.6 mL min^−1^ flow rate, and sulforaphane was detected at 196 nm using DL-sulforaphane standard (Sigma-Aldrich, St. Louis, MO, USA) for its identification and quantification (y = 3 × 10^7^ + 113,910; R^2^ = 0.9996; b = 27,200,000; LOD: 0.0040 mg mL^−1^; LOQ: 0.0133 mg mL^−1^). Results were expressed as g kg^−1^ dw. All the extraction procedures and analyses were performed in triplicate. Chromatograms and spectrums of identified compounds are shown in Figures S2 and S3.

### 2.4. Statistical Analysis

Two models were fitted to the data, consisting of three replicates for each time value and combination of factors. Firstly, the Peleg model was used, which is a kinetic model previously used in food byproduct extraction [31,32]. Its parameters are interpretable in terms of the kinetics of the extraction process providing insight on the process’ dynamics. On the other hand, an empirical polynomial model was fitted to the curves, concretely a degree 3 polynomial, which is called a cubic regression model. Such model has also been previously used for extraction procedures [33,34,35,36,37]. The cubic model does not aim at providing a mathematical kinetic model to explain the extraction process, in which parameters are interpreted as relevant kinetic quantities linked to the reactions. Instead, a cubic model offers a more flexible alternative for fitting, although its parameters are not directly interpretable in terms of the extraction’s dynamics. The latter is a drawback, but an advantage is that it allows for the application of the statistical theory of linear models. This approach enables the testing of the significance of effects and the assessment of estimation errors. Consequently, although a physical model is not obtained, the cubic model may provide data-based insights into the influence of several factors on process efficiency. Equations (1) and (2) describe the Peleg and cubic models, respectively:

(1) $$Peleg\;model:\;y\left(t\right)=y_{0}+\frac{t}{k_{1}+k_{2}t}$$

(2) $$\;Cubic\;model:\;y\left(t\right)=a_{0}+a_{1}t+a_{2}t^{2}+a_{3}t^{3}$$

where “y” represents the response variable (TPC, SFN) and t the time values (0, 2.5, 5, 7.5, 10, 15, and 20 min). Considering a full factorial design for the combinations of the following factor values: Tª = “25, 40, and 55 °C”; broccoli part = “Florets and Leaves”; solid/liquid ratio = “R1—1:25 g:mL and R2—2:25 g:mL”, separate curves were fitted to “y” given the t values. The coefficients were estimated from the experimental data using the least squares method and R^2^ value was computed. The observed values were represented in scatter plots together with the fitted models for all extraction conditions. All statistical analyses were performed with R software (2023).

## 3. Results

Results for obtained broccoli byproduct characterization are presented in Table 1. Broccoli tissues studied were rich in TPC and TAC, especially broccoli leaves, while broccoli florets were richer in SFN. Although some differences can be observed among the three methods developed to measure TAC, we can justify these differences as we analyzed TAC from different perspectives. With the FRAP method, we measured the ability of the extracts to reduce the iron, while with DPPH and ABTS methods were used to measure the ability of the extracts to scavenge free radicals both in methanolic and in aqueous media, respectively. No differences were shown in GL content. According to these initial values, comparable to previous findings in the same products, the US extractions were carried out to isolate these compounds depending on the selected variables.

Clear and concise organization, schematically shown in Figure 1, was required to carry out the experiment according to the studied variables. TPC, SFN, GLF, GLB, and ΣGL, depending on solid/liquid ratio, time, and temperature conditions during the UAE, are shown in Table 2 for florets and in Table 3 for leaves, for which the minimum and maximum values for TPC, SFN, and ΣGL are shown in Table 4

### 3.1. Variables according to Raw Material: Part of the Broccoli

As shown in Table 2 and Table 3, the TPC and SFN content varied according to the part of the broccoli used as extraction source. In fact, UAE extracts from broccoli leaves were among 5- and 12-fold higher in TPC than broccoli floret extracts, which varied according to the studied extraction conditions. By contrast, the extraction of sulfur compounds (GLS and SFN) was higher in broccoli florets than in leaves. For instance, the GLF, GLB, and ΣGL extraction was a 15–25% higher in florets than in leaves (Table 2 and Table 3). Similarly, this increase in SFN content obtained from UAE broccoli florets was from 2.5- to 4.5-fold compared to the leaf’s extracts obtained from the same plants. This behavior justifies that the variables that did not reach significant values cannot be modelled, as occurred with the obtained results for TPC in florets, SFN in leaves, and GLS in both parts of the broccoli byproducts analyzed. In fact, the concentrations of such biocompounds were not high enough to be modelled in the present work, for which they did not have a good adaptation to the studied models. This is the main limitation of the present research, as other previous researchers assessed [38,39,40]. In this sense, as R^2^ was lower than 0.5 for both statistical models of the cited variables, their representation is not shown in the present work, although obtained results from those analyses are shown in Table 2 and Table 3.

### 3.2. Operating Conditions during UAE of Broccoli in Leaves and in Florets: Ratio, Time, and Temperature

**Figure 2:**
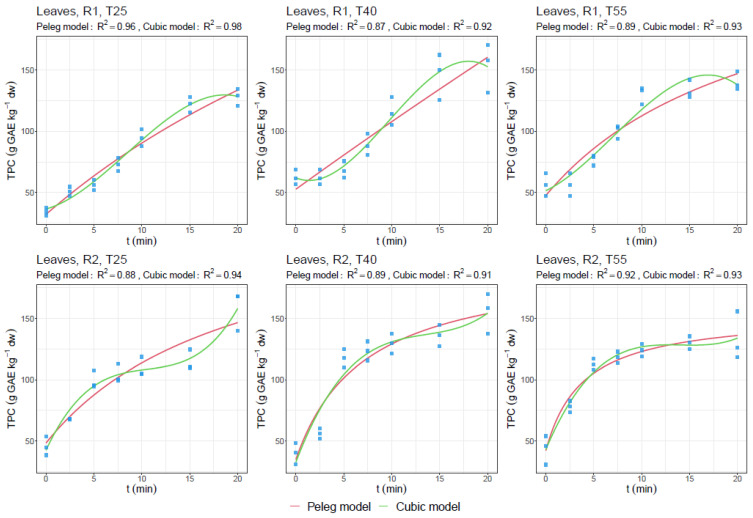
Experimental data (symbols) and fitted curves (Peleg and cubic models) for predicting total phenolic content (TPC) (g GAE kg^−1^ dw) in broccoli leaf byproduct solid/liquid ratios (R1—1:25 g/mL (up) and R2—2:25 g/mL (down)), time (0–20 min), and temperature (T25, T40, and T55; °C).

**Figure 3:**
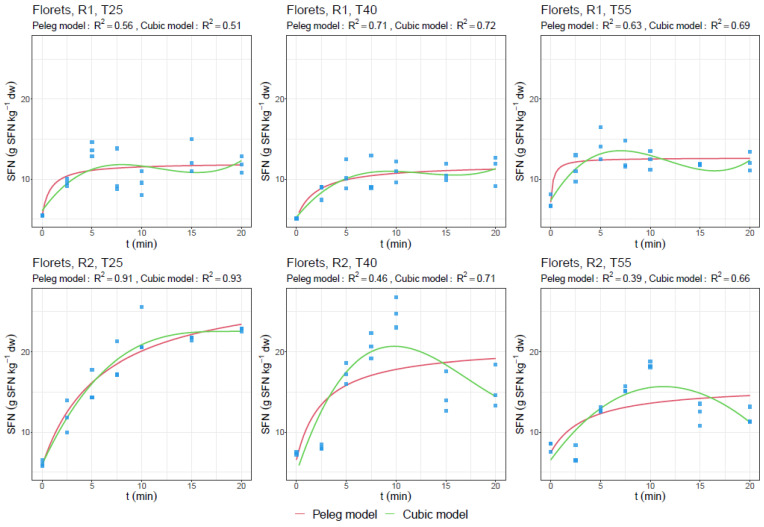
Experimental data (symbols) and fitted curves (Peleg and cubic models) for predicting sulforaphane content (SFN) (g kg^−1^ dw) in broccoli floret byproduct solid/liquid ratios (R1—1:25 g/mL (up) and R2—2:25 g/mL (down)), time (0–20 min), and temperature (T25, T40, and T55; °C).

Figure 2 and Figure 3 show the experimental data and fitted curves for predicting TPC in leaves and SFN in florets, respectively, during the extraction time at several temperatures (25, 40, and 55 °C), and at two solid/liquid ratios (R1—1:25 g/mL and R2—2:25 g/mL). 

Regarding the UAE of broccoli florets, the best results were reported by a ratio of 1:25 g/mL (R1). The highest TPC yield was shown after 12.5 min and 40 °C, being the optimum for time and temperature, with approximately 13 g GAE kg^−1^ dw. However, from the operative point of view, according to energy savings and optimum extraction of other phytochemicals, the use of a lower temperature (such as 25 °C) is recommended, assuming a slight decrease of 10% in TPC (11.7 g GAE kg^−1^ dw). Additionally, variables that did not fit studied models are also shown in Table 2, Table 3 and Table 4, which present a lower adherence because of the variability of the biological system and the low concentration found in such products to be modelled (<10 g kg^−1^ dw).

With regard to the UAE of broccoli leaves, the best results were reported for ratio 2:25 g/mL (R2). The highest TPC yield was obtained after 20 min and 25 °C, with 158.7 g GAE kg^−1^ dw of leaves, 12-fold more than from florets, as reported above. No great differences were found between the studied temperatures (i.e., 155.2 g GAE kg^−1^ dw was obtained at 40 °C), for which the use of a lower extraction temperature (25 °C) is also here recommended again according to energy saving. These results show the best adherence to studied models due to the high concentration of phenolic compounds present in this matrix (>100 g kg^−1^ dw). Regression coefficients of both models are shown in Table 5 and Table 6, according to the data shown in Figure 2 and Figure 3.

## 4. Discussion

The maximum TPC extracted is highly variable due to the wide range of selected extraction parameters used according to previous studies, which has been recently summarized as particle size, frequency, power, temperature, solvent, and time [2]. This makes it necessary to discuss our results based on the effect of each variable studied in the previous sections (Table 5 and Figure 2 and Figure 3).

It has been stated that leaves make up half of the entire broccoli plant’s biomass [2], and compared to broccoli florets or stalks, broccoli leaves contain higher TPC and, hence, higher TAC, chlorophylls, and vitamins (E and K), while having similar GLS content [3,41]. Furthermore, its accumulation depends on factors like genotype, variety, cultivar, growing conditions, developmental stage, postharvest handling, and, as it is shown, type of plant tissue, which can justify the higher accumulation of GLS and SFN in inflorescences [2]. Specifically in these experiments, GLF and GLB have been identified in both plant tissues, as also reported by previous authors such as Ares et al. [22].

According to previous reports, Wu et al. [42] performed the extraction of phenolic compounds from broccoli inflorescences throughout enzymatic and UAE (single and combined). They obtained an extraction yield of ~1.05 mg GAE g^−1^ fresh weight (fw) using a power 500 W for 30 min at 50 °C and ratio 1:7 g/mL. These values can be comparable to our data since 13 g GAE kg^−1^ dw is equivalent to ~1.4 mg GAE g^−1^ fw (ratio fw/dw of broccoli florets= 9.3, obtained from the weight before and after freeze-drying). In fact, these authors only obtained increased values (~1.7 mg GAE g^−1^ fw) throughout an enzymatic extraction (7.5 mg g^−1^ cellulase + 1 mg g^−1^ papain + 10 mg g^−1^ pectinase) and throughout the combination of both treatments (enzymatic and ultrasounds), whose result was a phenolic yield of ~1.85 mg GAE g^−1^ fw. Therefore, they concluded that the best extraction conditions for phenolic compounds from broccoli inflorescences was a ratio of 1:6.88 g/mL, 430.16 W, pH 6.06, and 54.47 °C [42]. Although these data are 34% higher than our results, this fact may be due to the plant material used, the growing conditions as well as the extraction parameters used, or the combination with enzymes. Hence, we recommended an extraction temperature in the present study, which leads to lower energy costs having a more efficient, environmentally friendly process.

In the case of GL extraction, both time and temperature did not affect to the extraction rate, which demonstrates that with shorter times and low temperatures, such as 2.5 min at 25 °C, efficient extraction can be achieved. The 1:25 ratio (R1) was again the most effective for this objective. The obtained values were 5.5, 5, and 10.2 g kg^−1^ dw of floret powder for GLF, GLB, and ΣGL, respectively. Nevertheless, the obtention of SFN from such extraction was around 15 g kg^−1^ dw after 5 min using the 1:25 ratio and 25 g kg^−1^ dw after 10 min using the 2:25 ratio (R2) and 25 °C or 40 °C.

González et al. [43] have also shown that ratio and time of extraction are essential factors in the recovery of phenolic compounds, isothiocyanates, or glucosinolates. Their highest recoveries of SFN from inflorescences were obtained with a solid/liquid ratio of 1:50, 80% ethanol in the extractant, and 70 min of extraction, which resulted in 0.566 g SFN kg^−1^ dw, 46.8 g GL kg^−1^ dw, and 4.054 g GAE kg^−1^ dw. These values are lower than our SFN values, and higher than our GL values reported in the present work. This fact demonstrates the importance of applying ultrasound as an extractant force during these procedures to enhance their extraction yield.

Accordingly, Shokri et al. [44] showed that the highest SFN yield was reached under an 18 kHz and 500 W UAE of broccoli florets for 7 min at 60 °C and a ratio of 7.5:25 g/mL with 1.73 g kg^−1^ dw. Furthermore, Mahn et al. [8] showed similar SFN values (1.18 g kg^−1^ dw) in blanched broccoli florets at 60 °C for 4 min of ultrasound-assisted blanching. In both cases, it was inversely proportional to the myrosinase activity measured. Therefore, it is remarkable how, in our present work, the obtained values for SFN and GLS content were ~16.6-fold higher than those obtained by Shokri et al. [44] and Mahn et al. [8] at higher temperatures and within less time. For this reason, we recommend using the freeze-dried product to enhance the extraction of these phytochemicals, even by using an UAE with bath instead of a probe to apply the ultrasound waves.

Regarding leaf extracts, previous works have already demonstrated that this part of the plant is richer in phenolic compounds than inflorescences (>1.60-fold) or stems (>1.59-fold) [45]. In this sense, Gudiño et al. [45] performed an UAE in a bath for 1 h at 45–50 °C with a ratio of 1:6 g/mL in 80% ethanol. They obtained 24.35 and 10.74 g GAE kg^−1^ dw for leaves and inflorescences, respectively [45]. Compared to our values (158.7 g GAE kg^−1^ dw and 13 g GAE kg^−1^ dw, for leaves and florets, respectively), it is appreciated, as phenolic compounds measured in inflorescences increased by ~21% while our extraction conditions (ratio of 2:25 g/mL water, 720 W, 35 kHz, 20 min, 25 °C) allowed for an increase in phenolics yield in broccoli leaves by ~6.5-fold regarding values reported by Gudiño et al. [45].

According to GL extraction, neither time nor temperature affected the extraction rate, which demonstrates again that short times and low temperatures can be recommended. The extraction at 25 °C during 2.5 min reported values of 5 g kg^−1^ dw of leaf powder for GLF and GLB and 10 g kg^−1^ dw for ΣGL, which is quite similar to broccoli florets. Once again, the R1 ratio (1:25) was the most effective. The SFN obtention from such extraction of broccoli leaves was around 5 g kg^−1^ dw for a 1:25 ratio and 3.5 g kg^−1^ dw for a 2:25 ratio after 5 min and at 25 °C, which is significantly lower (1.74- and 3.42-fold, respectively) than what was extracted in broccoli florets.

Thus, and according to the proposed models, high affinity to the fitted models in the case of TPC for leaves (Figure 2) and SFN for broccoli florets (Figure 3) can be appreciated, which is highly influenced by the concentration of such compounds in the tissues. In this regard, at higher amounts, the fitting to the proposed model is also high, while at lower amounts, the affinity to the predictive model decreases.

More recently, Cao et al. [23] established their optimal parameters for the UAE of phenolic compounds from broccoli leaves: a ratio of 1:36.35 g/mL, 49.5 °C, 31.4 min, and 383 W ultrasonic power. Under these parameters, the phenolic yield was 4.91 g kg^−1^, which is 31-fold lower than our obtained results. However, such notable variations can be due to the plant material, growing conditions, and extraction parameters used, among other factors. For example, the power applied by our equipment was 87% higher (720 W) than that applied by Cao et al. [23], so most of the time, results are quite difficult to compare.

Lastly, although no previous results have been found for the extraction of sulfur bioactive compounds from broccoli leaves, Liu et al. [7] studied other broccoli parts such as its seeds. They showed an SFN content from 14 to 18 g kg^−1^ under 500 W UAE during 20–40 min at 25 °C, ratio 1:10 g/mL, previously treated with microwaves and hydrolysis.

In this sense, according to the assessment of the results in the overall general context of the topic, in the present work, we described the behavior of the extraction of secondary metabolites presents in broccoli leaves and florets that are usually discarded. The concentration of the phytochemicals analyzed varied according to the ratio used and the time of extraction. These results fitted to the Peleg kinetic model, which has been used in similar studies [31,32], although our results seem to follow a cubic model, to which they are better adapted (>R^2^), as also reported by other researchers [33,37,46]. These facts are due to the lower concentrations found, as previously reported in several works [38,39,40]. Future studies must elucidate the use of mathematical and kinetic models that can justify this behavior in the UAE in broccoli byproducts.

## 5. Conclusions

The solid/liquid ratio influenced the extraction of SFN and GL in broccoli florets and leaves. It was confirmed higher sulfur bioactive compound contents in floret byproducts, while leaf byproducts were much richer in phenolic compounds, which were affected by the extraction time and ratio used. An extraction temperature of 25 °C is recommended for energy saving, since slight differences were found among the studied temperatures. Particularly, our proposed predictive models showed high adherence to the studied conditions, being highly fitted in the case of TPC extraction from broccoli leaves. The limitation of our study is related to the plant material, as agrotechnical and climatic conditions have a great influence on the differentiation of phytochemicals content of the tested plant tissue. Therefore, the extraction of bioactive compounds from broccoli byproducts has great potential to be improved and optimized according to the phytochemical to be extracted, the source of extraction, and the technology used for this purpose.

## Figures and Tables

**Table 1:** Characterization of obtained broccoli byproducts.

	Broccoli Florets	Broccoli Leaves
TPC (g GAE kg^−1^ dw)	7.17 ± 0.23	35.24 ± 0.81
TAC–FRAP (g TE kg^−1^ dw)	0.04 ± 0.00	11.44 ± 0.25
TAC–DPPH (g TE kg^−1^ dw)	0.10 ± 0.00	15.78 ± 1.01
TAC–ABTS (g TE kg^−1^ dw)	0.25 ± 0.03	25.05 ± 0.41
SFN (g kg^−1^ dw)	5.51 ± 0.07	2.52 ± 0.09
GLF (g kg^−1^ dw)	5.50 ± 0.11	5.01 ± 0.07
GLB (g kg^−1^ dw)	5.31 ± 0.08	5.12 ± 0.12
ΣGL (g kg^−1^ dw)	10.81 ± 0.12	10.13 ± 0.31
Moisture (%)	12.36 ± 0.36	8.80 ± 0.40

**Table 2:** Ultrasound-assisted extraction parameters and mean values of resulting response variables for broccoli florets.

Common Variables			Broccoli Florets				
Time (min)	Tª (°C)	Ratio (g/mL)	TPC (g GAE kg^−1^ dw)	SFN (g kg^−1^ dw)	GLF (g kg^−1^ dw)	GLB (g kg^−1^ dw)	∑GL (g kg^−1^ dw)
0	25	1:25	6.7 ± 0.6	5.5 ± 0.1	5.5 ± 0.0	4.9 ± 0.1	10.4 ± 0.1
0	40	1:25	7.3 ± 0.3	5.1 ± 0.0	5.6 ± 0.0	5.4 ± 0.2	11.0 ± 0.3
0	55	1:25	7.5 ± 0.4	7.1 ± 0.9	5.6 ± 0.0	5.4 ± 0.0	11.0 ± 0.0
0	25	2:25	4.5 ± 0.9	6.1 ± 0.4	2.9 ± 0.0	2.6 ± 0.1	5.5 ± 0.1
0	40	2:25	6.2 ± 0.8	7.4 ± 0.2	2.9 ± 0.0	2.8 ± 0.0	5.7 ± 0.0
0	55	2:25	6.4 ± 0.7	8.2 ± 0.6	2.9 ± 0.0	3.2 ± 0.2	6.1 ± 0.2
2.5	25	1:25	7.2 ± 0.3	9.6 ± 0.5	5.5 ± 0.1	4.9 ± 0.0	10.4 ± 0.1
2.5	40	1:25	5.4 ± 0.4	8.5 ± 0.9	5.6 ± 0.0	5.4 ± 0.1	11.0 ± 0.1
2.5	55	1:25	6.2 ± 1.0	11.2 ± 1.2	5.6 ± 0.0	5.5 ± 0.1	11.1 ± 0.1
2.5	25	2:25	5.4 ± 0.4	11.9 ± 1.7	2.9 ± 0.0	2.6 ± 0.4	5.5 ± 0.4
2.5	40	2:25	6.1 ± 1.0	8.2 ± 0.3	2.9 ± 0.0	2.8 ± 0.0	5.7 ± 0.0
2.5	55	2:25	6.5 ± 1.3	7.1 ± 0.9	2.9 ± 0.0	3.1 ± 0.1	6.0 ± 0.1
5	25	1:25	5.6 ± 0.3	13.7 ± 0.9	5.4 ± 0.0	5.3 ± 0.0	10.7 ± 0.0
5	40	1:25	5.8 ± 0.7	10.5 ± 1.1	5.5 ± 0.1	5.5 ± 0.1	11.0 ± 0.2
5	55	1:25	8.3 ± 0.1	14.3 ± 1.5	5.3 ± 0.1	5.1 ± 0.0	10.4 ± 0.1
5	25	2:25	6.7 ± 1.0	15.5 ± 1.6	2.9 ± 0.0	3.2 ± 0.1	6.1 ± 0.1
5	40	2:25	6.7 ± 1.3	17.3 ± 1.8	2.9 ± 0.0	3.1 ± 0.1	6.0 ± 0.1
5	55	2:25	8.4 ± 0.4	12.8 ± 1.3	3.0 ± 0.0	3.3 ± 0.1	6.3 ± 0.1
7.5	25	1:25	6.5 ± 0.2	10.6 ± 1.0	5.7 ± 0.0	5.9 ± 0.1	11.6 ± 0.1
7.5	40	1:25	8.4 ± 0.3	10.3 ± 1.5	5.7 ± 0.0	5.3 ± 0.2	11.0 ± 0.2
7.5	55	1:25	8.5 ± 0.7	12.7 ± 1.3	5.7 ± 0.0	5.6 ± 0.0	11.3 ± 0.0
7.5	25	2:25	6.1 ± 0.2	18.6 ± 1.5	3.0 ± 0.0	3.2 ± 0.1	6.2 ± 0.1
7.5	40	2:25	6.7 ± 0.6	20.7 ± 0.3	3.0 ± 0.0	3.1 ± 0.1	6.1 ± 0.1
7.5	55	2:25	7.7 ± 1.3	15.3 ± 1.3	3.0 ± 0.0	3.2 ± 0.1	6.2 ± 0.1
10	25	1:25	9.0 ± 0.5	9.5 ± 1.0	5.7 ± 0.0	5.7 ± 0.1	11.4 ± 0.1
10	40	1:25	10.4 ± 0.5	11.0 ± 1	5.7 ± 0.0	5.6 ± 0.2	11.3 ± 0.2
10	55	1:25	9.3 ± 0.3	12.4 ± 1	5.7 ± 0.1	5.4 ± 0.0	11.1 ± 0.1
10	25	2:25	5.6 ± 0.2	22.3 ± 2.0	3.0 ± 0.0	3.0 ± 0.2	6.0 ± 0.2
10	40	2:25	6.9 ± 1.1	24.9 ± 1.9	3.0 ± 0.0	3.0 ± 0.0	6.0 ± 0.0
10	55	2:25	6.9 ± 0.6	18.4 ± 0.4	3.0 ± 0.0	3.1 ± 0.1	6.1 ± 0.1
15	25	1:25	11.7 ± 0.2	12.7 ± 1.1	5.6 ± 0.0	5.6 ± 0.0	11.2 ± 0.0
15	40	1:25	13.0 ± 0.8	10.8 ± 1.1	5.5 ± 0.0	5.4 ± 0.1	10.9 ± 0.1
15	55	1:25	12.5 ± 0.7	11.8 ± 0.1	5.3 ± 0.3	5.7 ± 0.4	11.0 ± 0.7
15	25	2:25	5.8 ± 0.6	21.7 ± 0.2	2.8 ± 0.1	2.7 ± 0.1	5.5 ± 0.2
15	40	2:25	6.1 ± 1.1	14.7 ± 1.8	2.8 ± 0.0	2.9 ± 0.1	5.7 ± 0.1
15	55	2:25	7.8 ± 0.4	12.3 ± 1.2	2.8 ± 0.0	2.9 ± 0.1	5.7 ± 0.1
20	25	1:25	7.3 ± 0.3	11.8 ± 1.0	5.3 ± 0.0	5.3 ± 0.1	10.6 ± 0.1
20	40	1:25	8.6 ± 0.7	11.3 ± 1.2	5.3 ± 0.0	5.3 ± 0.1	10.6 ± 0.1
20	55	1:25	8.9 ± 0.1	12.2 ± 1.1	5.3 ± 0.1	5.2 ± 0.0	10.5 ± 0.1
20	25	2:25	5.7 ± 0.1	22.7 ± 0.2	2.8 ± 0.0	3.0 ± 0.1	5.8 ± 0.1
20	40	2:25	5.9 ± 0.1	15.5 ± 2.5	2.8 ± 0.0	3.0 ± 0.0	5.8 ± 0.0
20	55	2:25	6.2 ± 0.1	11.9 ± 1.0	2.8 ± 0.0	3.1 ± 0.0	5.9 ± 0.0

TPC: total phenolic content; SFN: sulforaphane; GLF: glucoraphanin; GLB: glucobrassicin; ∑GL: total glucosinolate content. Data shown are the average of three replicates (n = 3).

**Table 3:** Ultrasound-assisted extraction parameters and the mean values of resulting response variables for broccoli leaves.

Common Variables			Broccoli Leaves				
Time (min)	Tª (°C)	Ratio (g/mL)	TPC (g GAE kg^−1^ dw)	SFN (g kg^−1^ dw)	GLF (g kg^−1^ dw)	GLB (g kg^−1^ dw)	∑GL (g kg^−1^ dw)
0	25	1:25	34.3 ± 3.2	2.5 ± 0.7	5.0 ± 0.1	5.1 ± 0.1	10.1 ± 0.2
0	40	1:25	62.2 ± 6.1	2.5 ± 0.5	5.1 ± 0.0	5.2 ± 0.0	10.3 ± 0.1
0	55	1:25	56.4 ± 9.1	1.9 ± 0.1	5.0 ± 0.0	5.1 ± 0.0	10.1 ± 0.1
0	25	2:25	45.6 ± 7.7	2.4 ± 0.0	2.6 ± 0.1	2.7 ± 0.1	5.3 ± 0.1
0	40	2:25	40.0 ± 8.7	3.2 ± 0.4	2.6 ± 0.0	2.7 ± 0.1	5.3 ± 0.1
0	55	2:25	43.5 ± 11.8	1.9 ± 0.2	2.7 ± 0.0	2.8 ± 0.0	5.5 ± 0.1
2.5	25	1:25	50.9 ± 3.8	4.1 ± 0.0	5.0 ± 0.0	5.0 ± 0.0	10.0 ± 0.0
2.5	40	1:25	62.2 ± 6.1	3.7 ± 0.3	5.0 ± 0.0	5.0 ± 0.0	10.0 ± 0.1
2.5	55	1:25	56.4 ± 9.1	2.2 ± 0.1	5.1 ± 0.0	5.1 ± 0.0	10.2 ± 0.0
2.5	25	2:25	68.0 ± 0.2	2.9 ± 0.1	2.7 ± 0.0	2.6 ± 0.0	5.3 ± 0.1
2.5	40	2:25	56.2 ± 4.2	2.8 ± 0.5	2.7 ± 0.0	2.7 ± 0.0	5.4 ± 0.0
2.5	55	2:25	78.1 ± 4.6	2.4 ± 0.1	2.9 ± 0.0	2.9 ± 0.0	5.8 ± 0.0
5	25	1:25	56.0 ± 4.1	4.6 ± 0.0	5.0 ± 0.0	4.9 ± 0.0	9.9 ± 0.0
5	40	1:25	68.4 ± 6.7	3.6 ± 0.1	4.9 ± 0.0	5.0 ± 0.0	9.9 ± 0.1
5	55	1:25	77.0 ± 4.3	2.8 ± 0.2	4.9 ± 0.1	4.8 ± 0.0	9.7 ± 0.1
5	25	2:25	99.3 ± 7.2	3.0 ± 0.1	2.5 ± 0.0	2.6 ± 0.1	5.1 ± 0.1
5	40	2:25	117.5 ± 7.3	3.2 ± 0.1	2.5 ± 0.0	2.6 ± 0.0	5.1 ± 0.0
5	55	2:25	112.4 ± 4.5	2.9 ± 0.2	2.6 ± 0.0	2.7 ± 0.0	5.3 ± 0.0
7.5	25	1:25	72.8 ± 5.3	5.0 ± 0.0	5.0 ± 0.0	4.9 ± 0.0	9.9 ± 0.0
7.5	40	1:25	89.0 ± 8.8	3.4 ± 0.6	4.9 ± 0.1	4.9 ± 0.0	9.8 ± 0.1
7.5	55	1:25	100.1 ± 5.6	3.4 ± 0.7	5.0 ± 0.0	5.0 ± 0.0	10.0 ± 0.0
7.5	25	2:25	104.2 ± 7.6	3.1 ± 0.1	2.5 ± 0.0	2.5 ± 0.0	5.0 ± 0.0
7.5	40	2:25	123.4 ± 7.7	3.3 ± 0.5	2.5 ± 0.0	2.6 ± 0.0	5.1 ± 0.1
7.5	55	2:25	118.1 ± 4.7	3.4 ± 0.1	2.6 ± 0.0	2.6 ± 0.0	5.2 ± 0.0
10	25	1:25	94.6 ± 7.0	5.5 ± 0.0	4.9 ± 0.0	5.0 ± 0.0	9.9 ± 0.1
10	40	1:25	115.7 ± 11.4	4.3 ± 0.4	4.9 ± 0.0	4.9 ± 0.1	9.8 ± 0.0
10	55	1:25	130.2 ± 7.3	4.2 ± 0.3	4.9 ± 0.0	5.0 ± 0.0	9.9 ± 0.1
10	25	2:25	109.5 ± 8.0	3.5 ± 0.3	2.5 ± 0.0	2.5 ± 0.0	5.0 ± 0.0
10	40	2:25	129.6 ± 8.1	3.2 ± 0.1	2.6 ± 0.0	2.7 ± 0.0	5.3 ± 0.1
10	55	2:25	124.0 ± 5.0	4.0 ± 0.0	2.6 ± 0.0	2.7 ± 0.0	5.3 ± 0.1
15	25	1:25	122.1 ± 6.5	4.4 ± 0.5	5.0 ± 0.0	5.1 ± 0.0	10.1 ± 0.0
15	40	1:25	146.0 ± 18.9	4.7 ± 0.2	5.0 ± 0.0	5.0 ± 0.1	10.0 ± 0.1
15	55	1:25	133.7 ± 7.4	2.5 ± 0.3	5.0 ± 0.0	5.0 ± 0.1	10.0 ± 0.1
15	25	2:25	114.9 ± 8.3	3.3 ± 0.5	2.5 ± 0.0	2.6 ± 0.2	5.1 ± 0.2
15	40	2:25	136.0 ± 8.5	3.8 ± 0.1	2.6 ± 0.0	2.7 ± 0.2	5.3 ± 0.2
15	55	2:25	130.2 ± 5.2	3.2 ± 0.1	2.6 ± 0.0	2.7 ± 0.1	5.3 ± 0.1
20	25	1:25	128.2 ± 6.8	4.7 ± 0.6	5.0 ± 0.1	5.0 ± 0.0	10.0 ± 0.1
20	40	1:25	153.4 ± 19.8	3.2 ± 0.4	4.9 ± 0.0	4.9 ± 0.0	9.8 ± 0.0
20	55	1:25	140.4 ± 7.7	2.8 ± 0.3	5.1 ± 0.1	4.9 ± 0.0	10.0 ± 0.0
20	25	2:25	158.7 ± 16.2	2.6 ± 0.2	2.6 ± 0.0	2.5 ± 0.0	5.1 ± 0.0
20	40	2:25	155.2 ± 16.6	3.4 ± 0.3	2.6 ± 0.1	2.5 ± 0.0	5.1 ± 0.1
20	55	2:25	133.4 ± 19.7	3.0 ± 0.1	2.7 ± 0.1	2.6 ± 0.0	5.3 ± 0.1

TPC: total phenolic content; SFN: sulforaphane; GLF: glucoraphanin; GLB: glucobrassicin; ∑GL: total glucosinolate content. Data shown are the average of three replicates (n = 3).

**Table 4:** Minimum and maximum values of resulting response variables for broccoli floret and leaf byproducts.

Broccoli Byproduct	Ratio S/L (g/mL)	Tª (°C)	TPC (g GAE kg^−1^ dw)		SFN (g kg^−1^ dw)		∑GL (g kg^−1^ dw)	
			Min	Max	Min	Max	Min	Max
Florets	1:25	25	5.6	11.7	5.5	13.7	10.5	11.6
		40	5.4	13.0	5.1	11.5	10.9	11.3
		55	6.2	12.5	7.1	14.3	10.4	11.2
	2:25	25	4.5	6.7	6.1	22.7	5.5	6.2
		40	5.9	6.9	7.4	24.9	5.7	6.1
		55	6.2	8.4	7.1	18.4	6.0	6.3
Leaves	1:25	25	34.3	128.2	2.5	5.5	9.9	10.1
		40	62.2	153.4	2.5	4.7	9.8	10.3
		55	56.4	140.4	1.9	4.2	9.7	10.2
	2:25	25	45.6	158.7	2.4	3.5	5.0	5.3
		40	40	155.2	2.8	3.8	5.1	5.4
		55	43.5	133.4	1.9	4.0	5.1	5.7

**Table 5:** Regression coefficients and respective standard errors (in brackets), and the determination coefficients for predicting TPC in broccoli leaves using Peleg and cubic models.

Ratio	T (°C)	Peleg Model			R^2^	Cubic Model				R^2^
		y_0_	k_1_	k_2_		a_0_	a_1_	a_2_	a_3_	
R1	25	32.1242 (3.8302)	0.1465 (0.0258)	0.0025 (0.0012)	0.96	79.8496 (1.28)	150.8509 (5.8655)	−16.5087 (5.8655)	−20.353 (5.8655)	0.98
	40	52.7071 (6.9503)	0.1772 (0.0559)	4 × 10^−4^ (0.0027)	0.87	99.5657 (2.5244)	161.0544 (11.5682)	−3.1431 (11.5682)	−38.4214 (11.5682)	0.92
	55	47.5688 (6.5134)	0.1068 (0.0309)	0.0047 (0.0015)	0.89	99.1674 (2.2504)	144.5158 (10.3127)	−38.7141 (10.3127)	−23.6763 (10.3127)	0.93
R2	25	48.2699 (6.8495)	0.1021 (0.0312)	0.0051 (0.0015)	0.88	100.0213 (1.9958)	144.3157 (9.1457)	−17.6421 (9.1457)	42.9451 (9.1457)	0.94
	40	34.9605 (8.019)	0.0443 (0.0124)	0.0062 (8 × 10^−4^)	0.89	108.253 (2.9528)	160.2136 (13.5312)	−69.2244 (13.5312)	31.875 (13.5312)	0.91
	55	42.1358 (5.5329)	0.0346 (0.0086)	0.0089 (7 × 10^−4^)	0.92	105.6543 (2.0459)	116.6464 (9.3754)	−70.4337 (9.3754)	28.3497 (9.3754)	0.93

**Table 6:** Regression coefficients and standard errors (in brackets), and the determination coefficients for predicting SFN in broccoli florets using Peleg and cubic models.

Ratio	T (°C)	Peleg Model			R^2^	Cubic Model				R^2^
		y_0_	k_1_	k_2_		a_0_	a_1_	a_2_	a_3_	
R1	25	5.4566 (1.1569)	0.1265 (0.1518)	0.1518 (0.0345)	0.56	10.4803 (0.4774)	6.5165 (2.1876)	−4.7602 (2.1876)	4.2494 (2.1876)	0.51
	40	5.0718 (0.7891)	0.2945 (0.1735)	0.1471 (0.0242)	0.71	9.6195 (0.3067)	6.986 (1.4056)	−5.0749 (1.4056)	3.1211 (1.4056)	0.72
	55	7.1287 (0.9008)	0.0465 (0.1185)	0.1806 (0.0363)	0.63	11.6921 (0.318)	3.9105 (1.4571)	−6.0302 (1.4571)	5.4192 (1.4571)	0.69
R2	25	5.8323 (1.0622)	0.2655 (0.0665)	0.0436 (0.0044)	0.91	16.9584 (0.3884)	23.1796 (1.78)	−11.962 (1.78)	2.3361 (1.78)	0.93
	40	6.5492 (2.7517)	0.189 (0.1794)	0.0698 (0.0202)	0.46	15.5111 (0.7818)	11.0634 (3.5828)	−19.942 (3.5828)	4.6796 (3.5828)	0.71
	55	7.451 (1.7599)	0.4373 (0.4558)	0.1188 (0.0413)	0.39	12.2975 (0.5135)	6.6053 (2.3531)	−11.9192 (2.3531)	0.553 (2.3531)	0.66

## Data Availability

The original contributions presented in the study are included in the article and Supplementary Materials, further inquiries can be directed to the corresponding author.

## Supplementary Materials

The following are available online at https://www.mdpi.com/article/10.3390/foods13101441/s1, Figure S1: Photographs of broccoli florets (A and B) and broccoli leaves (C and D) obtained by scanning electron microscopy (SEM); Figure S2: Chromatograms obtained from GLS (A) and SFN analysis (B). In A: 1. GLF; 2. GLB. In B: 1. SFN; Figure S3: Spectrum of identified compounds: GLF (A), GLB (B), and SFN (C).
