# Supplementary material for: Ultrasound-Assisted Extraction of Bioactive Compounds from Broccoli By-Products
Source: Foods. 2024 May 7;13(10):1441. doi: 10.3390/foods13101441 (PMC11120188; doi:10.3390/foods13101441)
Supplement: Supplementary file 1 [file foods-13-01441-s001.zip › foods-2929300-supplementary.pdf]

A

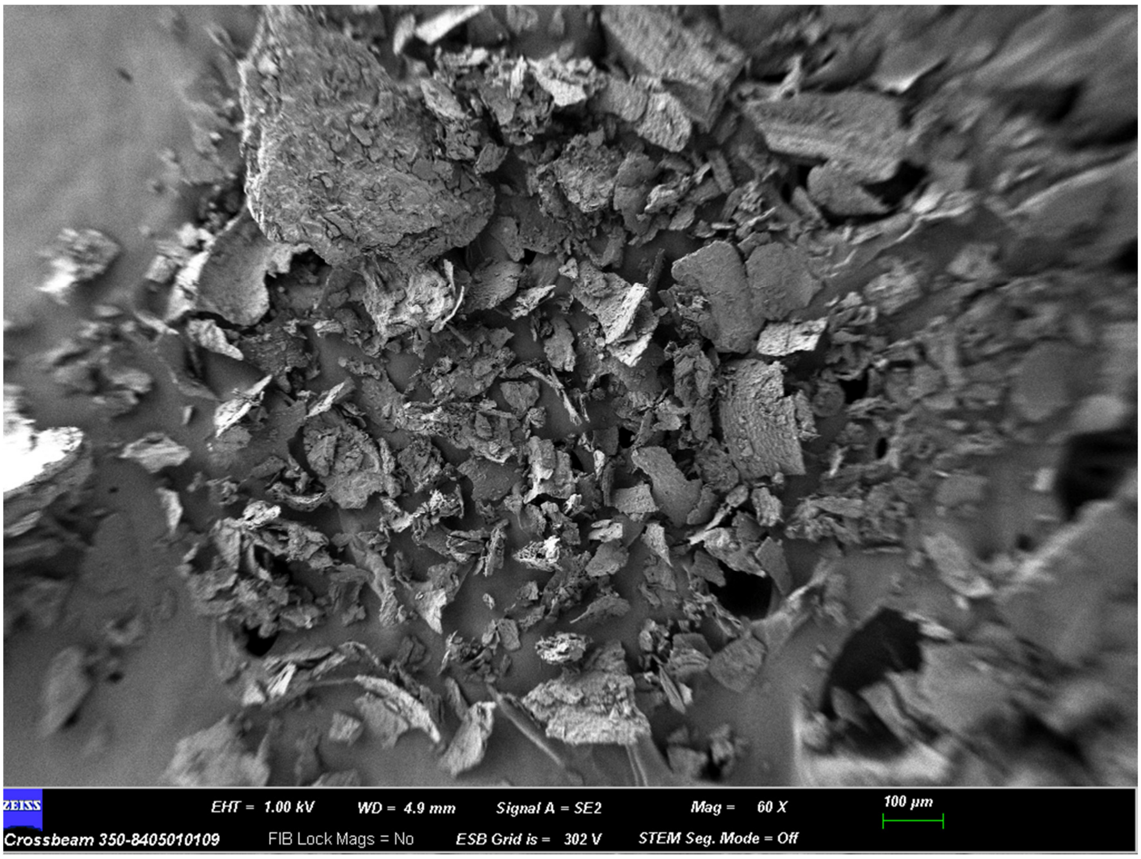
ZEISS
EHT = 1.00 kV
WD = 4.9 mm
Signal A = SE2
Mag = 60 X
100 µm
Crossbeam 350-8405010109
FIB Lock Mags = No
ESB Grid is = 302 V
STEM Seg. Mode = Off

B

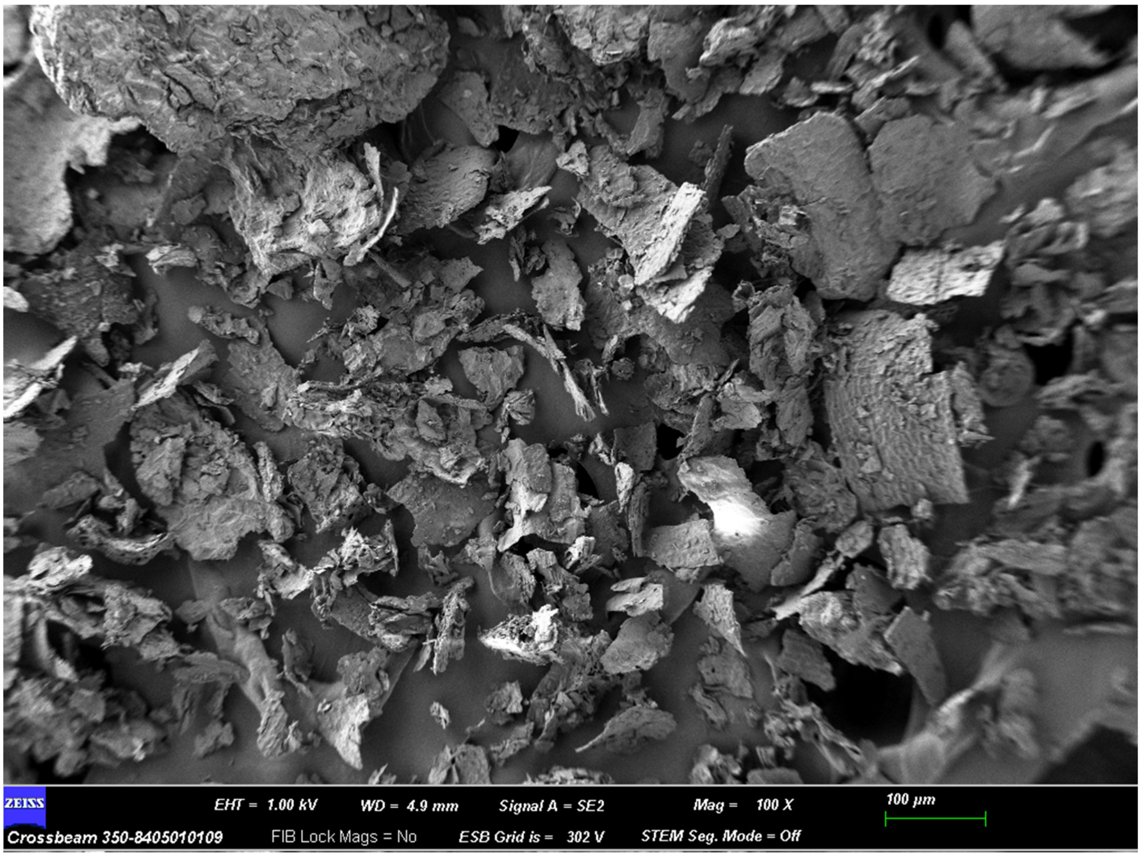
ZEISS
EHT = 1.00 kV
WD = 4.9 mm
Signal A = SE2
Mag = 100 X
100 µm
Crossbeam 350-8405010109
FIB Lock Mags = No
ESB Grid is = 302 V
STEM Seg. Mode = Off

C

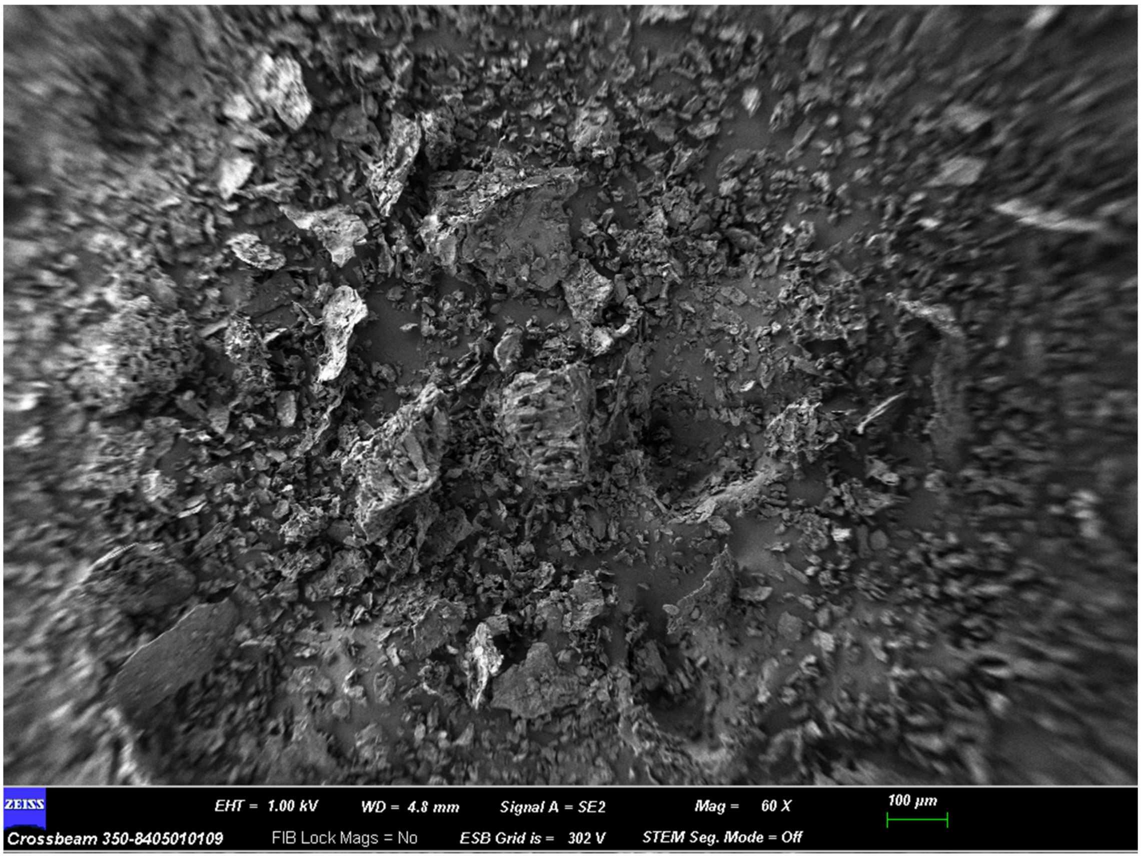

D

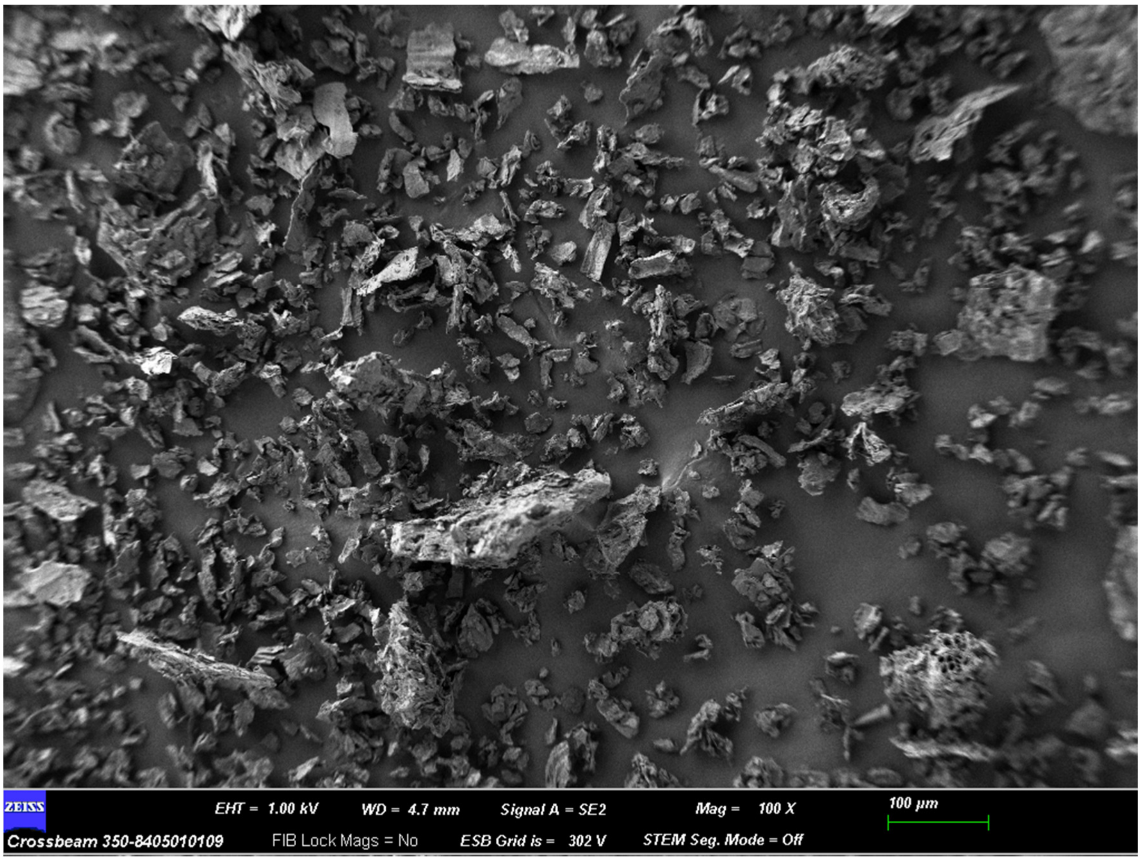

Figure S1. Photographs of broccoli florets (**A**,**B**) and broccoli leaves (**C**,**D**) obtained by scanning electron microscopy (SEM).

A

B

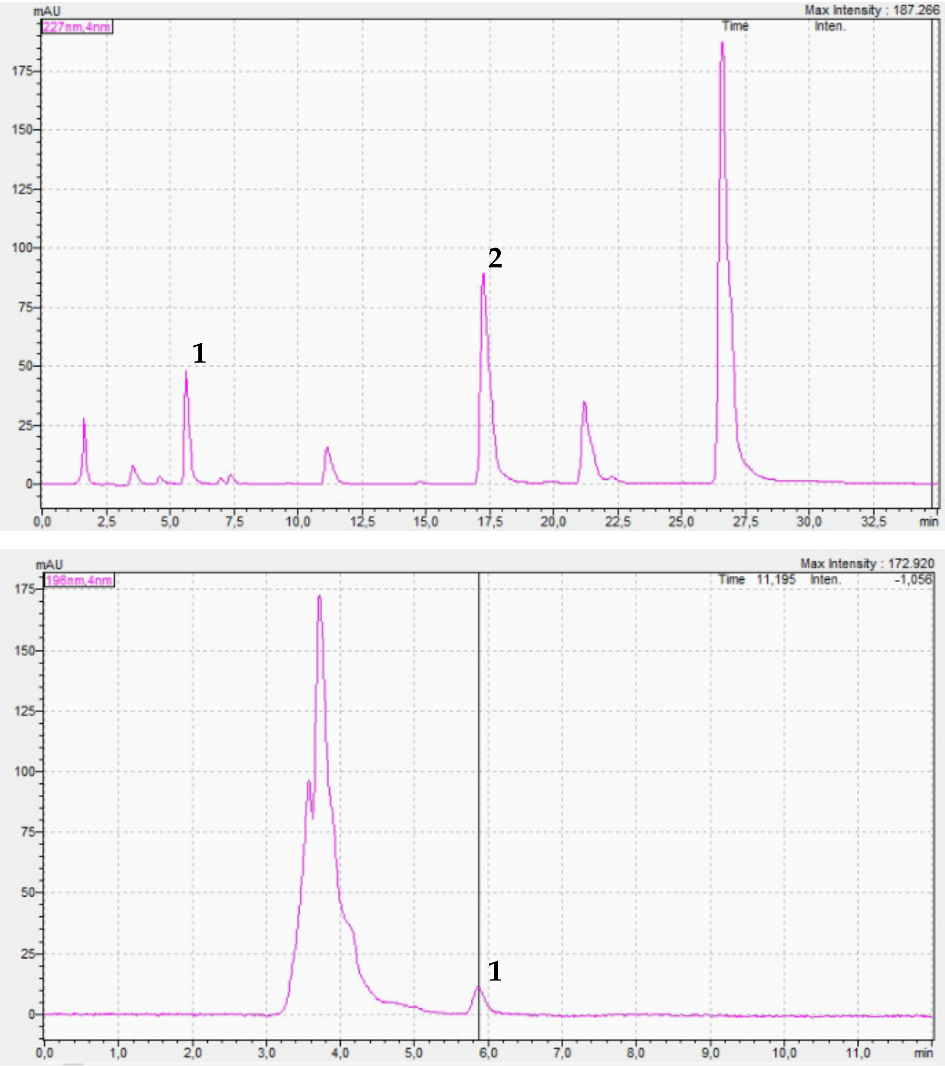

Figure S2. Chromatograms obtained from GLS (**A**) and SFN analysis (**B**). In A: 1. GLF; 2. GLB. In B: 1. SFN.

A

B

C

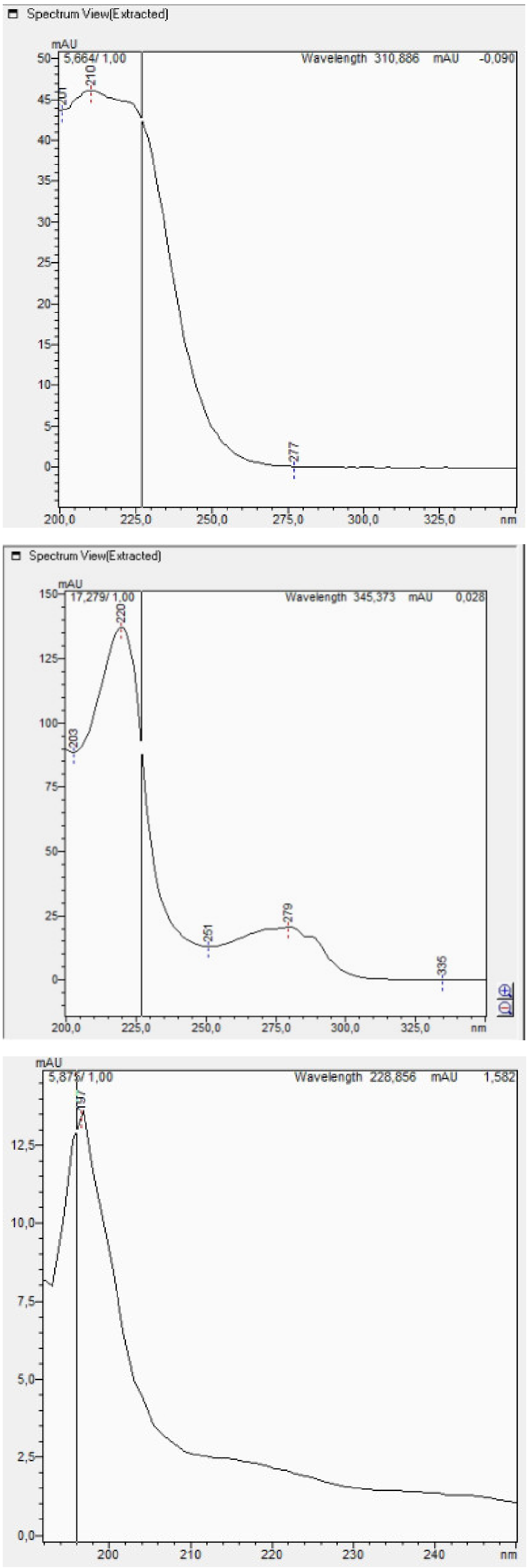

Figure S3. Spectrum of identified compounds: GLF (**A**), GLB (**B**), and SFN (**C**).
